# Circulating Tumour Cell Expression of Immune Markers as Prognostic and Therapeutic Biomarkers in Head and Neck Squamous Cell Carcinoma: A Systematic Review and Meta-Analysis

**DOI:** 10.3390/ijms21218229

**Published:** 2020-11-03

**Authors:** Karl Payne, Matthew Pugh, Jill Brooks, Nikolaos Batis, Graham Taylor, Paul Nankivell, Hisham Mehanna

**Affiliations:** 1 Institute of Cancer and Genomic Sciences, University of Birmingham, Birmingham B15 2SY, UK; j.m.brooks@bham.ac.uk (J.B.); N.Batis@bham.ac.uk (N.B.); p.c.nankivell@bham.ac.uk (P.N.); h.mehanna@bham.ac.uk (H.M.); 2 Institute of Immunology and Immunotherapy, University of Birmingham, Birmingham B15 2TT, UK; M.Pugh.1@bham.ac.uk (M.P.); g.s.taylor@bham.ac.uk (G.T.)

**Keywords:** circulating tumour cell, immune marker, head and neck cancer, HNSCC, immunotherapy

## Abstract

Rates of loco-regional recurrence and distant metastasis remain high among head and neck squamous cell carcinoma (HNSCC) patients, despite advancing cancer treatment modalities and therapeutic agents. One area that has generated considerable interest is the immune landscape of the tumour, heralding a wave of immune checkpoint inhibitors with notable efficacy in recurrent/metastatic HNSCC patients. However, HNSCC remains poorly served by biomarkers that can direct treatment in a personalised fashion to target the tumour heterogeneity seen between patients. Detection and analysis of circulating tumour cells (CTCs) in HNSCC has provided a previously unseen view of the metastasis forming cells that are potentially contributing to poor clinical outcomes. In particular, identifying CTC expression of phenotypic and druggable protein markers has allowed CTC sub-populations to be defined that hold prognostic value or are potential therapeutic targets themselves. The aim of this systematic review was to examine the role of CTC immune-marker expression as prognostic/therapeutic biomarkers in HNSCC by evaluating progress to date and discussing areas for future research. Our results highlight how few studies have been able to demonstrate prognostic significance of immune-marker expression in CTCs. As expected, the immune checkpoint PD-L1 was the most widely investigated marker. However, no studies evaluated CTC target immune marker expression in immunotherapy cohorts. Despite these findings, the data presented demonstrate promise that CTCs may be a source of future biomarkers for immunotherapy and will provide valuable information regarding the potential immune evasion of these metastasis forming cells.

## 1. Introduction

Up to 60% of patients with head and neck squamous cell carcinoma (HNSCC) can be expected to develop loco-regional recurrence [1] and 20–30% distant metastasis [2]. The majority of these recurrent/metastatic (R/M) patients are destined for palliative therapy only, in the form of chemotherapy +/- reirradiation and cytotoxic or targeted therapy if applicable [3]. Despite evidence that biomarker-led personalised treatment results in better patient outcomes [4], biomarkers in HNSCC remain underdeveloped. Tumour human papillomavirus (HPV) status (assessed via p16 expression) remains the most clinically significant biomarker in HNSCC [5], although a variety of emerging biomarkers remain under investigation [6]. However, the development of immune checkpoint inhibitors (ICIs) has heralded a wave of optimism in treating R/M HNSCC patients. Trials of ICIs in HNSCC, for example, the PD-1 inhibitors pembrolizumab [7] and nivolumab [8], have demonstrated response rates in the region of 20% and improved overall survival in R/M patient groups. These drugs act to block the PD-1 receptor on T-cells that when activated by PD-L1 from cells in the tumour cause immune suppression [9]. PD-1 inhibitors are now recommended as monotherapy for patients who have platinum-refractory R/M disease (progressing within 6 months of chemotherapy) [10]. Despite the success of these ICIs, 80% of R/M patients will still not respond to what is often highly morbid treatment. Biomarkers to direct treatment and predict response are therefore urgently required [4].

Research has sought to identify novel predictive and therapeutic biomarkers for ICIs, in addition to tumour PD-L1 expression [11]. These include genomic markers such as tumour mutational burden or microsatellite instability scores [11], or gene expression profiles to identify tumours likely to respond to treatment, such as the T-cell inflamed tumour subtype [12]. One potential source of novel immunotherapy-related biomarkers is a liquid biopsy—using a non-invasive blood test to detect circulating tumour DNA (ctDNA) or circulating tumour cells (CTCs)—which allows serial monitoring of treatment response [13,14]. CTCs are cells derived from the primary or metastatic tumour mass that are circulating within the blood and are responsible for the formation of secondary metastatic deposits at distant sites [15]. CTCs have been demonstrated as a source of potential diagnostic and prognostic biomarkers in HNSCC, with the ability to detect recurrence and monitor tumour dynamics [16]. Thus, utilising immune-marker expression on CTCs as prognostic and/or immunotherapy-related biomarkers is particularly promising. Our aim was to systematically review immune markers expressed on CTCs as potential biomarkers in HNSCC to assess their efficacy and the potential future avenues of research.

## 2. Results

### 2.1. Included Studies and Patient Clinical Demographic Data

**Figure 1:**
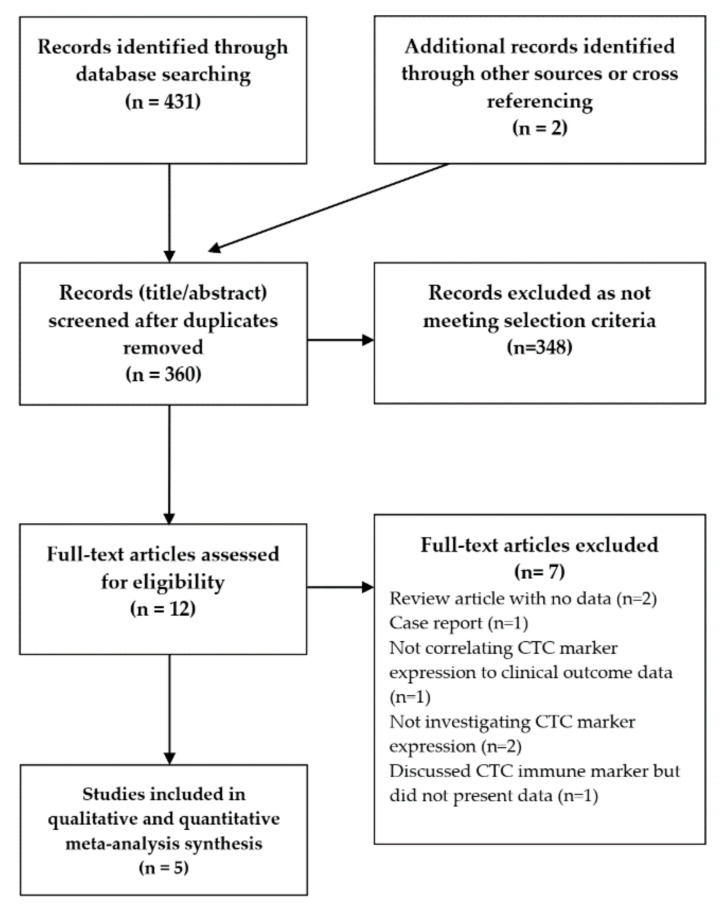
Study selection flowchart. CTC: circulating tumour cell.

After excluding reports not meeting the selection criteria (Figure 1), we identified five studies for qualitative analysis and quantitative meta-analysis (Table 1 and Table 2). Of note, one paper which was not included did discuss CTC immune marker expression (TLR4, TLR7, and TLR9) in HNSCC [17]. However, while the paper stated that these markers were not associated with progression free survival (PFS) or overall survival (OS), it did not present the actual data and so was excluded.

Considerable variability existed in the stage of disease between included studies, with two studies including stage I–IV HNSCC, two studies including locally advanced (stage III/IV) disease, and one study only including R/M patients. Documentation of treatment regimens within studies on the whole was acceptable. Two studies that assessed pre-treatment CTC status only stated that patients were “treatment naïve”. In the R/M cohort study, previous primary treatment was detailed but secondary/palliative treatment was not discussed. In the two papers with post-treatment data, both cohorts received cisplatin-based chemo-radiotherapy.

### 2.2. CTC Detection and Disease Stage

Assessing the correlation of CTC detection rate and disease stage between studies was not possible. In the two papers including locally advanced HNSCC patients, actual figures of “epithelial CTC positive” patients were not reported [18,22]. In these papers an EpCAM positive enrichment strategy was assumed to capture all epithelial expressing CTCs and gene expression values of this pooled cell group were normalised against a control value to assess overexpression of the target gene. In the R/M patient study, 80% (24/30) of patients were CTC positive [20], and in the stage I–IV patient studies 47.8% (11/23) and 63.6% (28/44) demonstrated detectable CTCs [19,21].

### 2.3. Immune Marker Expression and Disease Stage

Two studies compared CTC immune marker expression and stage of disease. Strati et al. reported no statistically significant relationship between CTC PD-L1 expression and stage of disease at pre-treatment or post-treatment timepoints (*p* = 0.340 and 0.328 respectively). Similarly, Economopoulou et al. reported no relationship between stage of disease and CTC IDO-1 expression at pre-treatment or post-treatment timepoints (*p* = 0.63 and 0.34, respectively). The remaining three studies did not compare CTC immune marker expression and stage of disease. Comparing CTC expression of all immune markers across four studies that reported actual numbers of positive CTCs we observed a statistically significant difference between the R/M and stage I–IV (including locally advanced disease) patient groups (*p* = 0.00327).

### 2.4. HPV Status

Study stratification based upon HPV status was not possible. Only one study clearly presented HPV status [19], with 47.8% (11/23) of patients being HPV positive. However, this study did not correlate HPV status to CTC PD-L1 expression. In another study, Strati et al. stated that of the CTC positive patients with known PD-L1 expression status, four were HPV positive and 3/4 of these demonstrated PD-L1 expressing CTCs [18]. However, they did not state the HPV status of the entire cohort or correlate this to clinical outcome variables. The three remaining studies did not discuss HPV status [20,21,22].

### 2.5. CTC Immune-Marker Expression as Tumour Therapeutic and Treatment Response Biomarkers

One study correlated tumour and CTC expression of the immune marker PD-L1 [20]. Twenty four of 30 R/M HNSCC patients (80%) were positive for CTCs, and among these 20 (83%) demonstrated PD-L1 expressing CTCs. In 23 patients with available tumour immunohistochemistry, 13 tumours expressed PD-L1, compared to 20 samples with PD-L1 expressing CTCs. In 10 patients (43.5%) the tumour and CTC PD-L1 expression were concordant (all PD-L1 positive cases). Whereas, in 10 patients CTCs expressed PD-L1, which did not match PD-L1 negative tumours. This equated to a positive and negative predictive value of 50% and 0% respectively. However, this paper did not correlate CTC PD-L1 expression and clinical outcome.

One study analysed pre-treatment CTC PD-L1 expression as a predictive marker of treatment response, with data from treatment outcomes from 73 treatment naïve patients all receiving chemo-radiotherapy [18]. Eighteen of the 59 CTC positive patients demonstrated CTC PD-L1 expression. Seven of these 18 (38.9%) achieved a complete response (CR) to treatment. Of those who were CTC PD-L1 negative at baseline (41/59), 27 (65.9%) achieved a complete response. Pre-treatment CTC PD-L1 expression was not correlated to treatment response (*p* = 0.085). However, post-treatment CTC PD-L1 expression was strongly correlated to treatment response (*p* = 0.001). Of 10 patients with overexpression of PD-L1 at the end of treatment, only 2 (20%) achieved a CR, whereas among 35 patients who were PD-L1 negative at the end of treatment, 28 (80%) demonstrated a CR. Thus, PD-L1 negative CTCs at the end of treatment was strongly associated with CR (OR: 16.00, 95% CI: 2.76–92.72, *p* = 0.002).

### 2.6. Pre-Treatment (Baseline) CTC Immune Marker Expression as a Prognostic Biomarker

**Figure 2:**
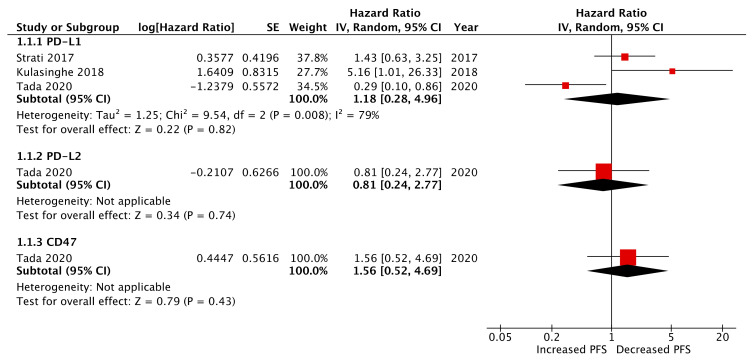
Forest plot and meta-analysis of pre-treatment CTC immune-marker expression as a biomarker of progression-free survival (PFS): Meta analysis performed on CTC PD-L1 expression (presented data may vary from Table 2 due to conversion formulae).

**Figure 3:**
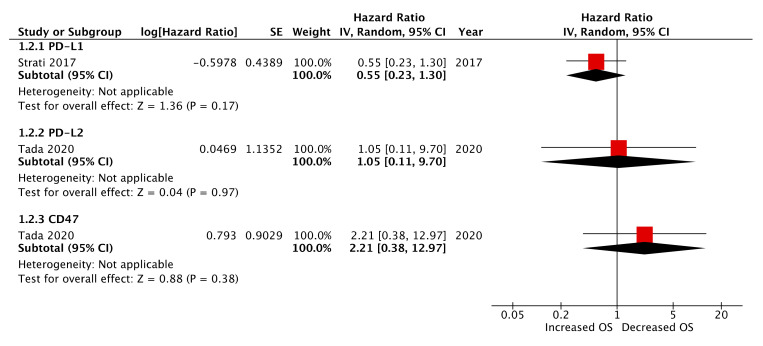
Forest plot of pre-treatment CTC immune-marker expression as a biomarker of overall survival (OS) (presented data may vary from Table 2 due to conversion formulae).

Four studies reported pre-treatment CTC immune-marker expression as prognostic biomarkers of PFS and/or OS (Figure 2 and Figure 3) [18,19,21,22]. Of these, three studies reported PD-L1 expression [18,19,21], one study PD-L2 expression [21], one study CD47 expression [21], and one study IDO-1 expression [22]. The relationship between pre-treatment CTC PD-L1 expression and PFS and OS appeared mixed. In a cohort of 94 patients assessed at baseline, Strati et al. reported 24 patients with PD-L1 expressing CTCs. In this cohort, CTC PDL1 expression was not associated with PFS (HR: 1.43, 95% CI: 0.63–3.25, *p* = 0.395) or OS (HR: 0.55, 95% CI: 0.23–1.30, *p* = 0.172). Kulasinghe et al. reported on a smaller cohort of 23 patients, of whom 11 were CTC positive and 6 patients exhibited PD-L1 expressing CTCs [19]. They correlated CTC PD-L1 expression to significantly decreased PFS (log-rank, HR: 5.159; 95% CI: 1.011–26.33, *p* = 0.0485). In contrast, Tada et al. demonstrated improved PFS and OS in patients with PD-L1 expressing CTCs (HR: 0.289, 95% CI: 0.0982–0.8643, *p* = 0.0346 and HR undefined, *p* = 0.0378, respectively). In the same paper CTC PD-L2 and CD47 expression was assessed, however, neither of these markers significantly correlated to PFS or OS. In this cohort of 44 patients, 64% (28) were CTC positive of which 11, 8, and 16 were PD-L1, PD-L2, and CD47 positive, respectively. Economopoulou et al. assessed CTC IDO-1 expression as a biomarker of PFS and OS in a cohort of 60 HNSCC patients. Twenty-four patients were IDO-1 positive pre-treatment, which correlated with improved PFS (log-rank, *p* = 0.01) but not OS (log-rank, *p* = 0.19). The association of IDO-1 pre-treatment expression and improved PFS was retained with multivariate analysis (cox regression, HR: 0.19, 95% CI: 0.03–0.46, *p* = 0.017). Meta-analysis of pre-treatment CTC PD-L1 expression among three studies demonstrated no association with PFS (HR: 1.17, 95% CI: 0.28–4.97, *p* = 0.83) (Figure 2) and a high heterogeneity between studies of 79%.

### 2.7. Post-Treatment CTC Immune Marker Expression

**Figure 4:**
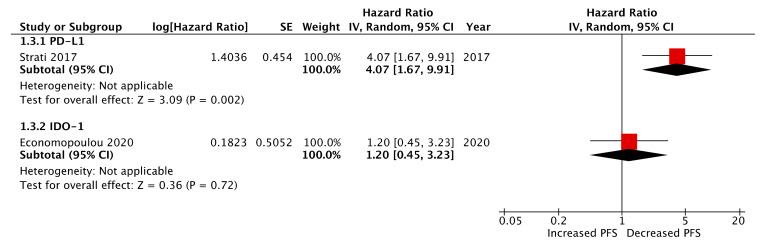
Forest plot of post-treatment CTC immune-marker expression as a biomarker of progression-free survival (PFS) (presented data may vary from Table 2 due to conversion formulae).

**Figure 5:**
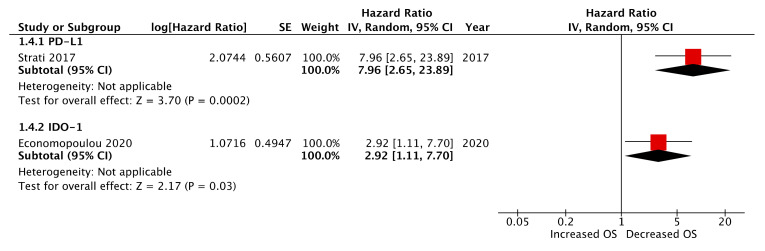
Forest plot of post-treatment CTC immune-marker expression as a biomarker of overall survival (OS) (presented data may vary from Table 2 due to conversion formulae).

Two papers evaluated CTC immune-marker expression at the post-treatment timepoint, of which both cohorts received cisplatin-based chemo-radiotherapy regimens [18,22]. In contrast to their findings pre-treatment, Strati et al. reported post-treatment CTC PD-L1 expression as a strong prognostic marker of deceased PFS and OS (log-rank, *p* = 0.001 and <0.001, respectively) [18]. Univariate Cox regression analysis demonstrated HRs of 4.07 (95% CI: 1.67–9.91) and 7.96 (95% CI: 2.65–23.89) for PFS and OS, respectively, and in both cases these far outweighed HR significance from other conventional prognostic clinicopathological variables (Figure 4 and Figure 5). In the aforementioned study by Economopoulou et al., IDO-1 expression assessed post-treatment was found to be a strong predictor of decreased OS but not PFS (contra to pre-treatment findings from this study) (Figure 4 and Figure 5). Multivariate analysis corroborated this association (cox regression, HR: 3.27, 95% CI: 1.03–2.05, *p* = 0.008).

### 2.8. Study Quality

The REMARK checklist was used to assess the quality of included studies (Table S2). Notable shortcomings across all studies was the description of patient recruitment including exclusion criteria, randomisation, and patient flow through the study. CTC enrichment strategies were generally well described, but blood collection strategies and specifically sample storage strategies were often vague. On the whole, univariate and multivariate statistical analysis were presented in a clear concise fashion, however, missing data or patient dropouts were poorly reported across studies.

## 3. Discussion

Since early research demonstrated proof-of-principle to identify CTC PD-L1 RNA transcript expression [23] and PD-L1 surface protein expression in HNSCC [24], several studies have sought to investigate multiple immune markers expressed by CTCs as potential biomarkers. In this systematic review, we identified five studies, all published within the last three years, which analysed small to large cohorts (range 24–113 patients) presenting correlation of CTC immune marker expression to clinical outcome data (PFS and OS). In sub-group meta-analysis, data from three studies evaluating pre-treatment CTC PD-L1 expression demonstrated no significant correlation to PFS. These findings are similar to a recent meta-analysis of tumour PD-L1 expression in HNSCC tumour tissue, which reported no significant difference in survival between the presence of PD-L1 positive and negative tumour cells in HNSCC [25]. To date, few studies have been able to assess CTC target immune-marker expression in cohorts of ICI treated patients. In a cohort of 40 melanoma patients treated with pembrolizumab, pre-treatment CTC PD-L1 expression was identified as an independent predictive biomarker of PFS [26]. More recently, Zhang et al. demonstrated novel data that monitoring of PD-L1 expressing CTCs and circulating tumour endothelial cells (CTECs) during anti-PD-L1 therapy predicted treatment response. Albeit in a small cohort of 16 patients, the presence of PD-L-1 expressing CTECs was associated with significantly worse PFS (*p* = 0.012) and notably this trend was not observed in CTCs [27]. The investigation of immune-marker expression on CTC sub-populations, for example with endothelial, stemness, or mesenchymal marker expression is particularly relevant to HNSCC, given evidence that these subtypes correlate with worse clinical outcomes [16]. Unfortunately, none of the studies identified in this review evaluated immune markers in CTC sub-groups (instead investigating epithelial expressing CTCs only) or as predictive biomarkers for ICI therapy stratification, instead evaluating clinical outcomes in largely heterogenous treatment groups.

However, evidence from two studies identified in this review indicates that post-treatment CTC immune-marker expression appears to have prognostic significance. Strati et al. and Economopoulou et al. demonstrated that CTC expression of the immune markers PD-L1 and IDO-1 at the post-treatment timepoint was associated with poor prognosis and reduced OS. IDO-1 is an interferon-induced enzyme associated with tumour immunosuppression [28]. Such prognostic significance of immune markers may represent an “immune-evasion” switch occurring in CTCs post-treatment leading to a poorer outcome. These findings corroborate those from Chikamatsu et al. who observed that CTC expression of the immune markers PD-L1, PD-L2, and CD47 were significantly correlated to each other (*p* = 0.0285, 0.0005, and 0.003, respectively) in R/M patients who had failed primary treatment, thus reinforcing the hypothesis of a CTC sub-population with an immune-evasion phenotype [29]. In patients treated with ICIs, the persistence of target immune-marker expression on CTCs of patients failing treatment has been highlighted as a means to identify resistance through “therapy escape” [30]. While at first glance the prognostic utility of pre-treatment CTC immune-marker (PD-L1) expression may appear poor, the benefit of liquid biopsies to enable serial testing of dynamic CTC immune-marker expression may provide a valuable tool in monitoring response to treatment, including ICI therapy. However, one factor to consider is that among the studies reviewed, the epithelial positive CTC detection rate was 48–80% (similar to other CTC studies in HNSCC [16]), thus raising questions as to the clinical applicability of this approach as a standardised biomarker assay. Further large cohort studies are required to investigate this. We found no evidence of serial CTC immune-marker monitoring in the post-treatment setting in HNSCC, however, evidence is growing in other cancer types [31]. Disease progression in patients treated with ICI therapy has been correlated to increased numbers of post-treatment PD-L1 expressing CTCs in lung cancer [32,33]. Strati et al. also clearly observed a significant trend for HNSCC non-responders (treated with chemo-radiotherapy) to demonstrate PD-L1 expressing CTCs. In this cohort, CTC PD-L1 negative patients were up to 16 times more likely to have a CR to treatment than PD-L1 positive patients, although this was not related to ICI therapy per se, it demonstrates a potential differing CTC immune profile in non-responders.

Patient stratification for ICI therapy is a continued dilemma in oncology [13]. Currently, tumour expression of PD-L1 is the only accepted means of patient stratification in HNSCC [10]. Expression of PD-L1 on tumour cells (TPS: tumour proportion score) has been shown to be a poor predictive marker of treatment response [34,35]. Instead, the composite proportion score/combined positive score (CPS), which assesses the ratio of PD-L1 expressing tumours and stromal cells, is a more accurate biomarker, and CPS ≥1 is currently recommended for patient selection [10]. However, CPS immunohistochemistry assays suffer from both inter-rater variability and inter-test variability from differing antibody epitopes [36]. In addition, tumour PD-L1 expression demonstrates both inter-patient (around 40% of HNSCC tumours express PD-L1 [25]) and intra-tumoural heterogeneity [37]. Thus, if CTCs can be shown as a reliable source of druggable immune markers they have potential to be a valuable clinical tool [11]. We identified only one study in HNSCC that correlated tumour and CTC PD-L1 expression [20]. CTC PD-L1 expression was seen to be a poor predictor of tumour PD-L1 expression, only being concordant in 50% of cases. Such findings of poor tumour PD-L1 expression predictive value in CTCs have been observed in other cancers [32,33]. There are several posited reasons for this discrepancy. Firstly, tumour PD-L1 expression is known to be both spatially and temporally heterogenous with considerable dynamic expression [37]. Of note, as seen by Chikamatsu et al., studies in other cancers such as non-small cell lung carcinoma have observed CTCs to be more often PD-L1 positive than tissue, up to two-fold [32]. This may represent PD-L1 intra-tumoural heterogeneity not detected in spatially biased single site tissue biopsies. Secondly, the complex relationship between CTCs and immune cells is still largely unknown. Such data pose an interesting question: Could the CTCs detecting intra-tumoural immune marker heterogeneity be a more accurate biomarker for immune therapy, or are the CTCs acquiring these changes in the circulation and do not accurately reflect the tumour? Evidence continues to grow that CTC expression profiles are fluid and dependent upon the micro-environment, with adaptation in the circulation in response to immune cell interactions [38]. PD-L1 expression may be one facet of this adaptation [29], however, as yet, this has not been directly investigated.

By assessing gene expression of pooled CTCs from enriched blood samples, one is able to interrogate increased numbers of markers than that of conventional immunofluorescent microscopy. However, it does not give you quantifiable numbers of single CTCs expressing the marker in question. Only one study in this review assessed expression of protein surface markers on CTCs [19]. Aside from correlating PD-L1 expression to PFS, a key message from this paper was the considerable heterogeneity of PD-L1 expression among CTCs from the same patient, even if over a small cohort. Of the six patients with PD-L1 expressing CTCs (range 1–20 CTCs per 3.75 mL blood), only in two patients were all detected CTCs PD-L1 positive. Regardless of using gene expression or surface marker PD-L1 positivity, all the papers identified in this review adopted a binary approach of patients being CTC PD-L1 negative or positive. However, as validated in tumour PD-L1 assessment, CTC immune-marker heterogeneity warrants a scale or percentage to quantify marker expression. Such an approach of low, medium, or high PD-L1 expression on single CTCs has been demonstrated to more accurately stratify patient response to ICI therapy [31,39], and may start to tease out associations with tumour expression and survival outcomes not currently seen in reported studies of HNSCC. The increasing utilisation of single-cell sequencing technologies to allow single-CTC gene-expression profiling is sure to answer several questions regarding CTC immune-marker expression in relation to intra-tumoural heterogeneity. It remains to be seen if predictive ICI gene-expression profiles from HNSCC tumours are also expressed in CTCs and if such multi-omic characterisation will enable more accurate patient treatment stratification.

## 4. Methods

A review protocol was designed a priori and approved by all authors. The Preferred Reporting Items for Systematic Reviews and Meta-Analyses (PRISMA) statement was followed when performing this systematic review and meta-analysis [40]. A study selection flowchart is detailed in Figure 1.

### 4.1. Search Strategy and Inclusion Criteria

Embase and Medline databases were searched using the Ovid interface. The following keyword search terms were used: (“head and neck cancer” or “head and neck squamous cell carcinoma” or “head and neck” or “HNSCC” or “SCCHN”) and (“circulating tumour cell” or “circulating tumor cell” or “CTC” or “neoplastic circulating cell”). Records were searched with no time limits up to June 2020. The results of this search were filtered to remove duplicates. Reference lists from identified papers were also examined to search for other relevant studies that may not have been identified by the primary search strategy.

Papers were included if they reported a recognised immune marker expressed on CTCs as a biomarker in HNSCC, this included correlation to treatment or clinical outcome variables as predictive or prognostic biomarkers and also comparison to tumour marker expression as a therapeutic biomarker. No minimum follow-up duration criteria were set. Only English language written papers were included. Case reports and conference abstracts were excluded but were screened to identify potentially eligible studies.

### 4.2. Study Selection and Data Extraction

Titles and abstracts of all identified studies were independently evaluated by two authors (KP and MP) after the removal of duplicate results. Studies selected were then judged according to their suitability for inclusion in the systematic review based on the eligibility criteria outlined. Any disagreement regarding inclusion was referred to a third author (PN). Data was independently extracted from each paper by the two reviewers and entered into a pre-designed excel proforma sheet. For each study we collected the following data points: author and year, stage of disease, size of patient cohort, method of CTC enrichment/detection, method of marker/gene identification, marker assessed, treatment timepoint at which CTCs were analysed, clinical outcome variable assessed with statistical data, and follow-up duration.

### 4.3. Data Analysis

Due to considerable heterogeneity between included studies, including the different immune markers investigated, a narrative synthesis was undertaken in the first instance. Data were presented graphically as forest plots and meta-analyses were performed using Cochrane RevMan.v5 software. As far as possible, log-rank hazard ratios (HRs) derived from Kaplan–Meier curves were presented. If HRs and confidence intervals (CIs) were not presented in the paper, the corresponding author was contacted for further data. If log-rank analysis was not available, univariate cox regression modelling HRs and CIs were utilised and analysed separately to log-rank data. As heterogeneity between studies was high (> 50%), a random effects model was used for meta-analysis of individual markers investigated in multiple studies (PD-L1). Quality assessment of included studies was undertaken using the Reporting Recommendations for Tumour Marker Prognostic Studies (REMARK) checklist [41,42] (Table S1).

## 5. Conclusions

In summary, we identify only a small number of studies examining immune-marker expression by CTCs in HNSCC with sufficient clinical outcome follow-up data. As expected, the majority of studies assessed the expression of the druggable target PD-L1. No clear trend was observed between PFS/OS and pre-treatment CTC immune-marker expression, although the multiple markers investigated by different studies make meta-analysis challenging. Despite these equivocal findings, post-treatment CTC immune-marker expression appears to hold prognostic significance and warrants further investigation. A key message is that, to date, no studies have investigated CTC immune-marker expression in HNSCC ICI therapy patient cohorts. If the translational line of site is utilising CTC liquid biopsies to risk stratify and monitor treatment response in immunotherapy patients, then future research should focus on these patient groups in HNSCC.

## Figures and Tables

**Table 1:** Data of clinicopathological variables, CTC enrichment/detection methods, and investigated marker/clinical outcome as extracted from included studies. (HNSCC: head and neck squamous cell carcinoma, PFS: progression free survival, OS: overall survival, MACS: magnetic assisted cell sorting, R/M: recurrent/metastatic, F/U: follow-up).

Author	Year	Tumour Site/Stage	No. of Patients in Cohort	Cohort Gender	Age (Years)	Tumour Site	CTC Enrichment Technique	Marker Detection Methodology	Immune Marker(s)	Timepoint of CTC Analysis	Outcome Measure (Median) and (Range) F/U
Strati et al. [18]	2017	Locally advanced HNSCC	113 (pre-treatment = 94, post-treatment = 54)	Pre-treatment: M = 75, F = 19, post-treatment: M = 37, F = 17	Pre-treatment: ≥65 = 40, <65 = 54, post-treatment: ≥65 = 25, <65 = 29	Pre-treatment: Oropharynx = 21, Other = 73, Post-treatment: Oropharynx = 13, Other = 41	Negative depletion (red cell lysis and CD45 MACS depletion) and EpCAM MACS enrichment	Gene expression using RT-qPCR	PD-L1	Pre-treatment and post-treatment	PFS and OS [18.9 months (0.2–54.9)]
Kulasinghe et al. [19]	2018	HNSCC stage I–IV	23	M = 17, F = 6	<60 = 10, >60 = 23	Oral cavity = 9, Oropharynx = 14	Microfluidic (ClearCell FX CTChip marker-independent) enrichment	Surface marker assessment with IF antibody	PD-L1	Pre-treatment	PFS [not stated]
Chikamatsu et al. [20]	2019	R/M HNSCC	30	M = 27, F = 3	Median 70.5 (range 53–86)	Oral cavity = 3, Nasopharynx = 1, Oropharynx = 3, Hypopharynx = 12, Larynx = 4, Paranasal sinuses = 6, Parotid gland = 1	Negative depletion (density centrifugation and CD45 MACS depletion)	Gene expression using RT-qPCR	PD-L1, PD-L2, CD47	R/M post-treatment	Biomarker of therapeutic PD-L1 target on tumour
Tada et al. [21]	2020	HNSCC stage I–IV	44	M = 41, F = 3	<66 = 21, ≥66 = 23	Nasal cavity = 3, Oral cavity = 4, Nasopharynx = 2, Oropharynx = 17, Hypopharynx = 14, Larynx = 4	CellSieve microfilter	Gene expression using RT-qPCR	PD-L1, PD-L2, CD47	Pre-treatment	PFS and OS [not stated]
Economopoulou et al. [22]	2020	Locally advanced HNSCC	113 (pre-treatment = 94, post-treatment = 54)	Pre-treatment: M = 75, F = 19, post-treatment: M = 37, F = 17	Pre-treatment: ≥65 = 40, <65 = 54, post-treatment: ≥65 = 25, <65 = 29	Pre-treatment: Oropharynx = 21, Other = 73, post-treatment: Oropharynx = 13, Other = 41	Negative depletion (red cell lysis and CD45 MACS depletion) and EpCAM MACS enrichment	Gene expression using RT-qPCR	IDO1	Pre-treatment and post-treatment	PFS and OS [27.16 months (2.3–69.3)]

**Table 2:** Hazard ratio (HR), 95% confidence interval (CI) and *p* value log-rank data extracted from included studies for progression free survival (PFS) and overall survival (OS). (* = univariate cox regression, ** = author derived from presented data).

Marker		PFS			OS		
	Pre-Treatment	HR	95% CI	*p* Value	HR	95% CI	*p* Value
PD-L1	Strati et al. 2017	1.43	0.63–3.25	0.39	0.55	0.23–1.30	0.17
	Kulasinghe et al. 2018	5.16	1.01–26.33	0.049			
	Tada et al. 2020	0.29	0.10–0.86	0.035	not stated	not stated	0.038
PD-L2	Tada et al. 2020	0.81	0.24–2.77	0.74	1.05	0.11–9.70	0.97
CD47	Tada et al. 2020	1.56	0.51–4.69	0.45	2.21	0.33–12.97	0.45
IDO1 *	Economopoulou et al. 2020	0.23	0.02–0.50	0.018	0.57	0.19–1.36	0.21
	**Post-treatment**						
IDO1 *	Economopoulou et al. 2020	1.20	0.25–3.23	0.75	2.92	1.11–7.70	0.011
PD-L1 *	Strati et al. 2017	4.07	1.67–9.91	0.002 **	7.96	2.65–23.89	0.0002 **

## Supplementary Materials

Supplementary materials can be found at https://www.mdpi.com/1422-0067/21/21/8229/s1.
