# Supplementary material for: Circulating Tumour Cell Expression of Immune Markers as Prognostic and Therapeutic Biomarkers in Head and Neck Squamous Cell Carcinoma: A Systematic Review and Meta-Analysis
Source: Int J Mol Sci. 2020 Nov 3;21(21):8229. doi: 10.3390/ijms21218229 (PMC7662307; doi:10.3390/ijms21218229)
Supplement: Supplementary file 1 [file ijms-21-08229-s001.pdf]

Table S1. The REMARK checklist[41] (table adapted with permission from Gurung et al.[42]).

**Introduction**
1a. Is the marker examined stated
1b. Study objectives stated?
1c. Pre-specified hypothesis stated?
**Materials and Methods**
Patients
2a. Are patient eligibility characteristics described
2b. Source of patients described – intervention?
2c. Source of patients described - control?
2d. Is exclusion criteria stated?
3a. Treatments described?
3b. How chosen – randomised, rule based, clinician choice?
Specimen characteristics
4a. Biological material used - intervention
4b. Biological material used - Control
4c. Preservation/storage described?
Assay methods
5a. Assay methods described?
5b. Assays performed blind to outcome?
Study design
6a. Retrospective sampling?
6b. Prospective sampling?
6c. Recruitment methods consecutive?
6d. Recruitment methods random?
6e. Matched controls?
6f. Study dates reported?
6g. Follow up times reported?
7a. All clinical endpoints defined?
8a. Candidate variables initially examined or considered for inclusion in models described
9a. Sample size given?
Statistical analysis methods
10a. Stats methods described?
10b. Model building/assumptions described?
10c. Missing data handling described?
11a. Marker values described?
11b. Cut off points reported?
**Results**
Data
12a. Flow of patients through the study reported?
12b. Number of dropouts and reasons reported?
12c. Subgroup analysis?
13a. Demographic characteristics reported?
13b. Missing values reported?
Analysis and presentation
14a. Show the relation of the marker to standard prognostic variables?
15a. Present univariable analyses showing the relation between the marker and outcome, with the estimated effect (eg, hazard ratio and survival probability).

16a. For key multivariable analyses, is the estimated effects reported - e.g. hazard ratio and confidence intervals for the marker
16b. For final model are all variables reported
17a. Among reported results, provide estimated effects with confidence intervals from an analysis in which the marker and standard prognostic variable are included, regardless of their statistical significance
18a. Are results from further investigations, such as checking assumptions, sensitivity analyses, and internal validation reported?
**Discussion**
19a. Are results interpreted in relation to the pre-specified hypotheses and other relevant studies.
19b. Are study limitations discussed?
20a. Discuss implications for future research
20b. and clinical value.

Table S2. The REMARK checklist [41] in array format to demonstrate study quality (adapted with permission from Gurung et al [42]). Included studies were assessed for each question with the possible responses of Yes (green), No (red), Partial (yellow), Unclear/Not Stated (pink), or not applicable (blue).

**Reference**	**21**	**24**	**23**	**25**	**22**
**Author / Year**	**Strati<br>2017**	**Kulasing<br>he<br>2018**	**Chikamats<br>u<br>2019**	**Tada<br>2020**	**Econom<br>opoulou<br>2020**
**INTRODUCTION**					
1a. Is the marker examined stated					
1b. Study objectives stated?					
1c. Pre-specified hypothesis stated?					
**MATERIALS & METHODS**					
2a. Are patient eligibility characteristics described					
2b. Source of patients described – intervention?					
2c. Source of patients described - control?					
2d. Is exclusion criteria stated					
3a. Treatments described?					
3b. How chosen – randomised, rule based, clinical choice?					
4a. Biological material used - intervention					
4b. Biological material used - Control					
4c. Preservation/storage described?					
5a. Assay methods described?					
5b. Assays performed blind to outcome?					
6a. Retrospective sampling?					
6b. Prospective sampling?					
6c. Recruitment methods consecutive?					
6d. Recruitment methods random?					
6e. Matched controls?					
6f. Study dates reported?					
6g. Follow up times reported?					
7a. All clinical endpoints defined?					
8a. Candidate variables initially examined or considered for inclusion in models described					
9a. Sample size given?					
10a. Stats methods described?					
10b. Model building/assumptions described?					
10c. Missing data handling described?					
**RESULTS**					
11a. Marker values described?					
11b. Cut off points reported?					
12a. Flow of patients through the study reported?					

12b. Number of dropouts & reasons reported?
12c. Subgroup analysis?
13a. Demographic characteristics reported?
13b. Missing values reported?
14a. Show the relation of the marker to standard prognostic variables?
15a. Present univariable analyses showing the relation between the marker &outcome, with estimated effect (e.g. HR &survival probability).
16a. For key multivariable analyses, is the estimated effects reported - e.g. hazard ratio & confidence intervals for the marker
16b. For final model are all variables reported
17a. Provide estimated effects with CI from analysis where marker & standard prognostic variable are included
18a. Are results from further investigations, such as checking assumptions, sensitivity analyses, & internal validation reported?
19a. Are results interpreted in relation to the pre-specified hypotheses & other relevant studies.
**DISCUSSION**
19b. Are study limitations discussed?
20a. Discuss implications for future research
20b. & clinical value.
